# Mechanisms Controlling Arsenic Uptake in Rice Grown in Mining Impacted Regions in South China

**DOI:** 10.1371/journal.pone.0108300

**Published:** 2014-09-24

**Authors:** Junhui Li, Fei Dong, Ying Lu, Qiuyan Yan, Hojae Shim

**Affiliations:** 1 College of Natural Resources and Environment, South China Agricultural University, Guangzhou, China; 2 Agricultural Bureau of Xiangfen County, Shanxi Province, Xiangfen, China; 3 Institute of Wheat Research, Shanxi Academy of Agricultural Sciences, Linfen, China; 4 Department of Civil and Environmental Engineering, Faculty of Science and Technology, University of Macau, Macau SAR, China; United States Department of Agriculture, Agricultural Research Service, United States of America

## Abstract

Foods produced on soils impacted by Pb-Zn mining activities are a potential health risk due to plant uptake of the arsenic (As) associated with such mining. A field survey was undertaken in two Pb-Zn mining-impacted paddy fields in Guangdong Province, China to assess As accumulation and translocation, as well as other factors influencing As in twelve commonly grown rice cultivars. The results showed that grain As concentrations in all the surveyed rice failed national food standards, irrespective of As speciation. Among the 12 rice cultivars, “SY-89” and “DY-162” had the least As in rice grain. No significant difference for As concentration in grain was observed between the rice grown in the two areas that differed significantly for soil As levels, suggesting that the amount of As contamination in the soil is not necessarily the overriding factor controlling the As content in the rice grain. The iron and manganese plaque on the root surface curtailed As accumulation in rice roots. Based on our results, the accumulation of As within rice plants was strongly associated with such soil properties such as silicon, phosphorus, organic matter, pH, and clay content. Understanding the factors and mechanisms controlling As uptake is important to develop mitigation measures that can reduce the amount of As accumulated in rice grains produced on contaminated soils.

## Introduction

Arsenic (As) is a carcinogenic metalloid ubiquitous in the environment, and is obtained from natural and anthropogenic sources [1], [2]. Anthropogenic activities such as metal mining and smelting, the use of As-containing pesticides, herbicides, wood preservatives, feed additives, and irrigation with As-rich groundwater, have resulted in elevated As levels in soil [1], [3]. The transfer of As in soil-plant systems represents one of the principal pathways for human exposure to As [4]. A recent cohort study [5] showed that daily consumption of 500 g cooked rice containing As content above 200 µg/kg can give rise to genotoxic effects in humans.

Rice is the staff of life for 3 billion people, predominantly in Asia [6], contributing over 70% of the energy and 50% of the protein provided by their daily food intake [7]. China is the world’s top rice producer, producing 36.9% of the world’s rice yield on 22.8% of world rice cropping area [8], and a top rice consumer with more than 60% of the Chinese population relying on rice as a dietary staple [2]. Unfortunately, among grain crops, rice is particularly efficient in As accumulation as it is generally cultivated in flooded paddy fields where As is more soluble and available to plant uptake [9], [10].

Some studies have revealed that As concentrations in rice grains were associated with the As concentrations in irrigated groundwater and/or soil [11]–[13], although rice grain can accumulate relatively large amounts of As even from soils not contaminated by As [14]. Others have shown that As in rice does not directly depend on total As concentration in soil and groundwater but may be due to various other factors controlling As solubility, bioavailability and uptake in the soil-rhizosphere-plant system [15]–[17]. Redox chemistry is one of the predominant factors controlling As speciation and solubility in soil [15]. Arsenic in paddy soil is taken up by plant roots via macro-nutrient transporters; arsenate via the phosphate transporters, and arsenite via silicon transporters [3], [18]. Iron (Fe), through forming iron plaque on rice root surfaces, has strong influence on As-uptake by rice roots [19]. The soil physiochemical properties, e.g., redox condition, pH, organic matter, soil texture, Fe and Mn oxides, and sulfur, also affect the solubility and bioavailability of As [15], [20]. In addition, the As concentration in various rice tissues varies between rice genotypes [21]. Understanding the genetics associated with grain As concentration is crucial for developing mitigation measures to counter the problem of food-chain contamination by As.

Arsenic is a natural component of Pb, Zn, Cu, and Au ores. Therefore, As is commonly found in soils in mine impacted regions at elevated levels, posing a risk to human and ecosystem health [1]. Paddy rice is one of the most important grain crops in South China [22]. The present study is centered on the Lechang and Renhua Pb-Zn mining regions located in the north of the Guangdong Province, South China. In this study, As levels for soil, root surface, root, straw, and grain were obtained so that grain As levels could be reviewed in regard to both soil and straw As levels. The objectives of this study were: to characterize concentrations of As in rice grains grown in the mining regions with elevated and non-elevated soil As levels, to explore the transfer of As from (rhizosphere) soil through the plant to grain, to identify rice cultivars with low As accumulation in grains, and to understand how the iron and manganese plaque on root surfaces, other macro- and micro-nutrients within the paddy soil and other edaphic properties influence As uptake, assimilation and redistribution, in order to develop potential strategies for reducing As accumulation in rice grains.

## Materials and Methods

### Ethics statement

No specific permits were required for the described field studies. No specific permissions were required for these locations. We confirm that the location is not privately-owned or protected in any way. We confirm that the field studies did not involve endangered or protected species.

### Study area

**Figure 1:**
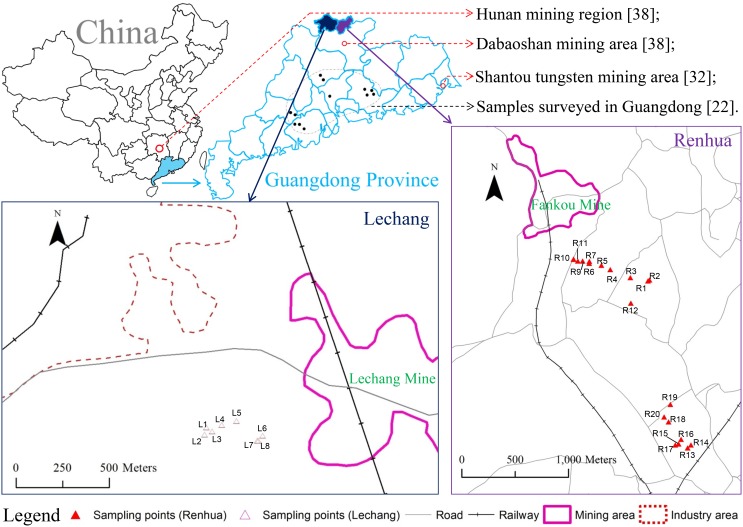
Location map of the study area and distribution of sampling sites.

The present study was conducted in two mining regions in the northern part of Guangdong Province, China (Fig. 1). This research area has a humid subtropical climate with a long-term average annual temperature of 19.6°C and an average annual precipitation of 1,522 mm [23]. The Fankou Pb-Zn mine is an extremely large mine located in Renhua County (Fig. 1). It is geologically situated in the northern part of the central Guangdong Hercynian trough of the South China parageosyncline. The mine was put into production in September 1968, and currently produces 4,500 t of ore per day. Major ore minerals in the Fankou ore mine are pyrite, sphalerite, and galena [24], [25]. The mine is classified as a submarine hydrothermal spring effusion type lead/zinc mine, which is relevant to reformed sedimentary rock. [25], [26]. Lechang Pb-Zn mine is located in Lechang County (Fig. 1). The major ore minerals are sphalerite, galena, pyrite, and chalcopyrite. As a conventional underground operation, this ore mine was opened in 1959 and is still in operation with a cover area of 1.5 km^2^ and produces of 250,000 tons of waste rocks and 30,000 tons of tailings per year occupying respective 8,300 and 60,000 m^2^ [23]. The ore of both mines is finely disseminated and complicated and the flotation technology to treat the core is so complex that it is difficult to remove contaminants and make use of the wastewater [27]. The surrounding paddy fields were seriously affected by the continuing year-round irrigation usage of untreated mining wastewater lifted from mines and filtrated from tailings [23]. The rice cropping system in the study area is double-season rice.

### Soil and plant samples collection

A total of 28 soil and 28 rice plant (including root, straw, and grain) samples were collected at maturity from 28 paddy fields within or adjacent to the two Pb-Zn mining areas, i.e., eight from Lechang and twenty from Renhua (Fig. 1). The fields were chosen primarily to reflect different rice cultivars being commonly grown by local farmers in these regions. The fields were not irrigated and drained 5 days prior to harvest. Soil samples, 0–15 cm depth, were collected from the base of the rice stem using a soil auger at harvest. At the same time, individual plants of twelve commonly grown cultivars of rice, i.e., Shanyou (SY)-82, SY-86, SY-89, SY-122, SY-162, SY-428, Tianyou (TY)-10, Meixiangzhan (MXZ)-2, Mabei-Youzhan (MBYZ), Diyou (DY)-162, Jinyou (JY)-118, and Fengyou (FY)-998, were collected from the sites where soil samples were taken (Table S1). Composite soil and plant samples were derived by mixing sub-samples from 5 random sites within 25 m^2^ per paddy field [22]. The six hybrid SY cultivars were genetically related in that they were all bred using Zhenshan 97A as the female parent. An entire single plant was dug up from 5 sites per each of the 28 fields. However, with the majority of the cultivars being grown only in Renhua or Lechang (three were grown in both), genotypic effects and regional effects were not distinct, but confounded. We therefore analyzed relationships between genotypic and environmental data across the two regions.

### Sample preparation

After harvesting, collected plants were washed thoroughly in tap water, followed by deionized water, before extracting the iron plaque from fresh root surfaces using dithionite-citrate- bicarbonate (DCB) as described by Liu et al. [28]. After DCB extraction, plants were separated into their respective tissue components (ear, straw, and root) with stainless steel scissors, weighed to determine fresh weight, oven-dried at 80°C for 72 h, then weighed again (dry weight). Dry spikelets were dehusked by hand and divided into grain and husk. The oven-dried root, straw, and grain samples were powdered using a model MM200 ball mill (Retsch, Germany). Soil samples were air-dried, crushed to pass through a 2 mm nylon sieve (10-mesh), and homogenized. The sub-samples were then ground with an agate grinder to pass through a 150 µm nylon sieve (100-mesh).

### Sample analyses

For soil digestion, 0.2 g soil samples were weighed into quartz glass tubes and 5 mL of 12.0 mol/L hydrochloric acid plus 5 mL of 15.2 mol/L nitric acid added, then left to stand overnight at room temperature before being digested on the block digester at 100°C for 1 h, then at 120°C for 1 h, and finally at 140°C for 4 h [22]. For digestion of plant tissues (root, straw, and grain), 0.2 g of grain samples, and 0.1 g of rice root and straw samples, were weighed into 50-mL polypropylene digest tubes and 2 mL of nitric acid added and left to stand overnight. Then 2 mL of hydrogen peroxide was added, and the samples digested using a microwave oven at 50% power (approximately 600 watts). The temperature was raised to 55°C held for 10 min, then to 75°C held for 10 min, and finally to 95°C for 30 min, and then allowed to cool to room temperature [22]. Total As concentrations in solution were determined by hydride generation atomic fluorescence spectrometry (AFS-8130, Beijing). Quality assurance and quality control of metal analyses were carried out by using duplicates (10% of the samples), reagent blanks and standard reference materials (National Environmental Monitoring Centre of China). The recoveries of As in standard reference materials were within ±10% of recommended values, and the relative standard deviation of duplicate measurements was less than 10%.

Selected soil properties, including soil pH, organic matter (OM), available phosphorus (AP), available silicon (ASi), cation exchange capacity (CEC), electrical conductivity (EC), and iron and manganese oxides, were analyzed according to the standard methods recommended by the Soil Science Society of China [29]. Free Fe and Mn, i.e., the bioavailable pool, were extracted by sodium sulfite-sodium citrate-sodium bicarbonate (DCB, mixture of 0.03 mol·L^−1^ Na_3_C_6_H_5_O_7_·2H_2_O, 0.125 mol·L^−1^ NaHCO_3_ and 0.5 g Na_2_S_2_O_4_) solution and determined by flame atomic absorption spectrophotometer (FAAS) (Hitachi Z-5300).

Iron plaque on fresh root surface was digested by DCB. For root digestion, 3 g mixed root sample per paddy was weighed into quartz glass tubes (100 mL) and steeped in 30 mL of DCB solution for 1 hr at 25°C before being transferred into quartz glass tubes (100 mL). The root surface As, Fe, and Mn digested by DCB were measured by AFS and FAAS respectively.

### Calculation of As transfer factors

Transfer factors (TFs) were determined using the expression C_p_/C_s_, where C_p_ is the concentration of As in straw or grain and C_s_ is the concentration of As in corresponding soil or straw.

Straw/soil transfer factors (S^traw^/S_oil_ TFs) = Straw As concentrations/Soil As concentrations.

Grain/soil transfer factors (G^rain^/S_oil_ TFs) = Grain As concentrations/Soil As concentrations.

Grain/straw transfer factors (G^rain^/S_traw_ TFs) = Grain As concentrations/Straw As concentrations.

### Statistical analyses

Descriptive analyses were conducted with SPSS V13.0 for Windows. Principal component analysis (PCA), based on the correlation matrix, was carried out with XLStat-Pro 7.5.2 software, used as a Microsoft Excel plug-in. A probability level of p<0.05 was considered as significant difference.

## Results and Discussion

### Arsenic concentrations in soils

Soil characteristics are presented in Table 1. Arsenic concentrations in the surface paddy soil ranged from 3.7 to 120 mg·kg^−1^ with an average of 30.4 mg·kg^−1^ in Pb-Zn across the two mining areas in our survey (Table 1). Although uncommon, cultivated paddy soils in Hunan Province, China have been found to contain up to 1,226.5 mg·kg^−1^ [30]. The As concentration of soils in this survey would be in the lower part of the range, compared with other mining impacted arable land, e.g., the Hengyang Pb-Zn mine area in Hunan Province (with an average of 253 mg·kg^−1^) [31], the Chenzhou Pb-Zn mine area in Hunan Province (405.7 mg·kg^−1^) [30], the Shantou abandoned tungsten mine region in Guangdong Province, China (129 mg·kg^−1^) [32], and the Rodalquilar Au-(Cu-Pb-Zn) mining district in Almería province, Southeastern Spain (180 mg·kg^−1^) [33].

**Table 1:** Descriptive statistics of soil properties of Renhua and Lechang.

Characteristics	Probability	Renhua (n = 20)		Lechang (n = 8)		Total (n = 28)
		Mean±SD	Range	Mean±SD	Range	Mean±SD
pH	0.0096	4.8±0.4	*4.4–*6.0	6.7±0.7	5.9–**7.6**	5.4±1.0
As-soil (mg·kg^−1^)	<0.0001	10.2±4.2	*3.7–*20.0	80.8±25.9	53.7–**120.3**	30.4±35.2
Fe_2_O_3_ (mg·kg^−1^)	0.93	9.3±4.3	*3.6–*21.3	26.0±4.7	21.3–**35.6**	14.1±8.8
Mn_2_O_3_ (mg·kg^−1^)	<0.0001	17.8±14.0	*7.9–*62.1	214.8±100.4	94.8–**361.3**	74.1±104.7
AP (mg·kg^−1^)	0.012	26.5±14.9	*9.2–* **57.5**	17.1±5.5	10.4–25.0	23.8±13.5
ASi (mg·kg^−1^)	0.013	53.3±13.8	*29.8–*88.1	100.3±26.7	73.1–**150.0**	66.7±28.1
CEC (cmol·kg^−1^)	0.91	6.6±1.1	*4.9–*8.4	16.4±1.2	14.8–**18.3**	9.4±4.6
OM (mg·kg^−1^)	0.61	30.7±5.6	*18.3–*43.7	47.0±6.6	37.4–**57.3**	35.4±9.5
EC (µs·cm^−1^)	0.0054	106.9±47.1	*49.6–*210	460.1±110.2	307–**631**	207.8±176.4
Sand (%)	0.39	31.3±4.6	*17.4–*36.4	26.4±5.9	18.8–**37.4**	29.9±5.4
Silt (%)	0.040	50.3±4.7	*42.7–* **63.6**	49.5±1.9	46.4–52.0	50.1±4.1
Clay (%)	0.010	18.3±2.3	*14.7–*23.9	24.1±4.7	16.2–**30.6**	20.0±4.1

Results are presented as arithemic mean ± SD; probability indicates the differences between Renhua and Lechang; n represents Number of samples; the *italic* number represents the minimum value of the characteristic in all the 28 surveyed samples; the **bold** number represents the maximum value of the characteristic in all the 28 surveyed samples.

Mean As concentrations in soils collected from Renhua were 10.2 mg·kg^−1^ (Table 1), similar to previously reported background concentration of 10.4 mg·kg^−1^ in Guangdong surface soils [34] and the median surface soil As concentrations in the paddy fields in Guangdong [22]. In contrast, soil As concentrations collected from Lechang were elevated, averaging 80.8 mg·kg^−1^, 8-fold higher compared to that of Renhua (Table 1). All eight soil samples collected from Lechang had As concentrations higher than the maximum allowable concentration (35 mg·kg^−1^ for soil with pH ≤5.5, 30 for soil with pH 5.5–6.5, 25 for soil with pH 6.5–7.5, and 20 for soil with pH ≥7.5) for agricultural soil in accordance with the Chinese Environmental Quality Standard for Soils [35]. Soil As concentrations exceeding 40 mg·kg^−1^ may be harmful to exposed organisms [36], and soil As exceeding 100 mg·kg^−1^ poses a severe risk to the pregnant women and their offspring [32], [37]. All of the eight samples from Lechang were over 40 mg·kg^−1^ As, two of which were over 100 mg·kg^−1^ As in this survey, indicating severe As contamination in soils around the Lechang Pb-Zn mine.

### Arsenic accumulation and translocation in tissues of rice plant

The overall mean total concentration of grain As was 0.26 mg·kg^−1^ (Table 2), which is comparable to field-collected unpolished rice from non-mining-impacted fields in Guangdong (0.29 mg·kg^−1^, n = 12, rice cultivar Peizha-Taifeng) [22] and Hunan mining region (0.30 mg·kg^−1^, n = 22) [38] (Fig. 1). In contrast, the average grain As in this survey was much higher than the field-collected unpolished rice from Dabaoshan mining areas in Guangdong (0.19 mg·kg^−1^, n = 95) [38] yet around half the grain As concentration reported in field-collected brown rice from Shantou tungsten mining area in Guangdong (0.56 mg·kg^−1^, n = 33) [32] (Fig. 1). All of the presently surveyed rice grains possessed As concentrations >0.17 mg·kg^−1^, exceeding Chinese maximum contaminant levels (MCLs) of 0.15 mg·kg^−1^, irrespective of As speciation [39], indicating that rice from this region would be a significant source of dietary As for the population. Chinese standards for As in rice are probably the strictest in the world, which have been designed to protect a nation with high rice intakes [38]. Compared to the global ‘normal’ range of 0.08–0.20 mg·kg^−1^ for As concentration in rice grain [12], 25 out of these 28 samples exceeded the ‘normal’ range. The mean As concentrations for these rice grain samples were much higher compared to that in rice from U.S. and Europe (both 0.198 mg·kg^−1^) [12].

**Table 2:** Descriptive statistics of rice plant accumulation and transfer factors.

Characteristics	Probability	Renhua (n = 20)		Lechang (n = 8)		Total (n = 28)
		Mean±SD	Range	Mean±SD	Range	Mean±SD
TF (soil-straw)	0.0047	0.36±0.18	0.13–**0.90**	0.079±0.041	*0.018–*0.13	0.28±0.20
TF (straw-grain)	0.62	0.084±0.30	*0.032–* **0.17**	0.057±0.023	0.036–0.11	0.076±0.030
TF (soil-grain)	0.0030	0.028±0.013	0.012–**0.057**	0.0037±0.0010	*0.0020–*0.0047	0.021±0.016
As-root (mg·kg^−1^)	0.49	25.6±12.5	*11.1–* **59.4**	35.0±9.4	24.8–52.0	28.3±12.3
As-straw (mg·kg^−1^)	0.029	3.3±1.3	*1.6–*6.9	5.8±2.8	2.1–**10.4**	4.0±2.1
As-grain (mg·kg^−1^)	0.37	0.25±0.051	0.18–**0.39**	0.28±0.067	*0.17–*0.38	0.26±0.06
As-DCB (mg·kg^−1^)	0.013	25.4±14.4	*6.4–*59.4	70.7±28.9	27.7–**101.8**	38.3±28.2
Mn-DCB (mg·kg^−1^)	<0.0001	25.6±22.5	*7.2–*93.4	189.1±109.4	75.6–**341.0**	72.3±95.5
Fe-DCB (g·kg^−1^)	0.044	29.7±11.4	*4.3–* **48.6**	36.3±5.0	26.5–44.6	31.6±10.4

Results are presented as arithemic mean ± SD; probability indicates the differences between Renhua and Lechang; n represents Number of samples; the *italic* number represents the minimum value of the characteristic in all the 28 surveyed samples; the **bold** number represents the maximum value of the characteristic in all the 28 surveyed samples.

It has been demonstrated that different rice cultivars showed significant differences for concentrations of As in straw, husk and grain [12], [19]. While we did not detect significant differences for rice grain As among the Lechang varieties, we did find difference between rice cultivars harvested from Renhua (Table 3). Although the genetic differences for As-root and As-straw were not significant, the cultivars SY-89 and DY-162 showed the lowest As concentrations for all tissues when grown in Renhua (Table 3), while the cultivar SY-122 showed the highest or second highest concentrations for all three tissues in both Renhua and Lechang. What makes this especially interesting is that, though the soils within each site were not significantly different for As concentration, the SY-122 with the highest tissue As was grown in a field with relatively lower soil As per site. Despite the fact that Lechang was higher than Renhua for As in soils, higher As concentrations of soil, DCB extracts, and straw, the As concentration in grains from the two sites was comparable (0.28 and 0.25 mg·kg^−1^) (Table 2). When data were analyzed among just the varieties grown in both Renhua and Lechang, As concentrations in grain were again comparable, i.e., SY-428 0.27 (Renhua)-0.25 (Lechang) mg·kg^−1^, MBYZ 0.24–0.29, and SY-122 0.39-0.32, in spite of their having more As in soil, DCB extracts, and straw in samples from Lechang compared to Renhua (Table 3). Although the mean soil As concentration in Renhua was lower compared to the national soil background, the rice grain As concentrations exceeded the Chinese MCLs, i.e., rice grain can accumulate relatively large amounts of As even from soils having very low level of As. Williams et al. [41] also reported that there were elevated grain As concentrations even with background soil levels. It is clear that the amount of As added by contamination to soil is not necessarily the overriding factor controlling the As concentration in rice grain. Other researchers [11], [12] reported that the high As levels in rice were associated with As-contaminated irrigation water. As uptake by rice mainly depends on As availability rather than total As in soil [14].

**Table 3:** Cultivar means for each parameter as observed in Renhua and Lechang.

Location	Cultivar	S^traw^/S_oil_	G^rain^/S_traw_	G^rain^/S_oil_	As-root	As-straw	As-grain	As-DCB	Mn-DCB	Fe-DCB	As-soil
		TF	TF	TF	mg·kg^−1^	mg·kg^−1^	mg·kg^−1^	mg·kg^−1^	mg·kg^−1^	g·kg^−1^	mg·kg^−1^
Renhua	SY-428	0.19±0.060b	0.11±0.053a	0.022±0.017b	22.6±11.2a	2.9±1.0a	0.27±0.052bc	25.4±7.8b	10.5±3.2a	28.0±4.4ab	16.0±6.8a
	MXZ	0.34±0.035ab	0.083±0.019a	0.028±0.0058ab	26.0±7.5a	2.8±0.4a	0.230.036cd	19.1±8.9b	35.3±30.3a	32.6±8.8ab	8.2±0.6b
	SY-162	0.37±0.10ab	0.086±0.015a	0.031±0.0047ab	22.2±5.2a	3.1±0.7a	0.26±0.016bc	24.9±16.3b	39.5±46.7a	29.8±19.9ab	8.7±0.8b
	MBYZ	0.57±0.46a	0.080±0.047a	0.034±0.010ab	38.1±30.1a	3.6±2.2a	0.24±0.0029bcd	14.5±5.6b	24.9±15.7a	32.9±13.9ab	7.2±2.0b
	TY-10	0.37±0.11ab	0.094±0.047a	0.037±0.027ab	19.5±5.3a	2.7±1.5a	0.22±0.012bcd	31.2±5.7b	31.9±11.4a	30.8±0.4ab	8.3±6.5ab
	SY-86	0.35±0.21ab	0.058±0.037a	0.016±0.00052b	30.0±14.7a	4.8±3.0a	0.22±0.00093bcd	57.5±2.7a	17.0±4.7a	38.0±6.1a	13.7±0.4ab
	SY-122	0.70a	0.081a	0.057a	48.6a	4.8a	0.39a	6.4b	16.5a	4.3b	6.9ab
	SY-89	0.21ab	0.084a	0.018b	11.1a	2.2a	0.19d	26.5b	21.5a	41.9a	10.8ab
	DY-162	0.23ab	0.079a	0.018ab	14.6a	2.3a	0.18d	23.5b	16.1a	23.1ab	9.9ab
	JY-118	0.40ab	0.064a	0.025ab	23.7a	4.8a	0.31ab	17.4b	18.9a	17.3ab	12.1ab
Lechang	SY-428	0.039±0.018c	0.077±0.026a	0.003±0.00062b	29.8±6.8b	3.6±2.2a	0.25±0.090a	68.2±33.1a	228.0±116.0a	37.4±1.3a	96.7±36.0a
	SY-82	0.12±0.010a	0.039±0.0043a	0.005±0.00011a	28.7±2.3b	8.6±2.6a	0.33±0.064a	64.8±52.4a	213.2±177.3a	35.6±12.8a	72.5±15.5a
	MBYZ	0.052bc	0.061a	0.003ab	43.0ab	4.7a	0.29a	67.0a	111.1a	35.7a	89.0a
	SY-122	0.12ab	0.038a	0.005a	52.0a	8.3a	0.32a	65.6a	75.6a	35.2a	68.3a
	FY-998	0.10ab	0.044a	0.005a	38.6ab	5.6a	0.25a	98.4a	215.4a	36.4a	53.7a

Results are presented as arithemic mean ± SD; means within a row for a certain genotype grown in Lechang or Renhua followed by different letters are significantly different at the 0.05 level; the comparisons are based on estimated marginal means.

Similar to the total As in soils, the DCB-extracted As concentrations from the root surfaces were highly variable from one paddy field to another, and there was an approximate 3-fold difference in mean DCB-extracted As concentration between Lechang and Renhua (Table 2). Interestingly, SY-122 as one of the three cultivars grown in both locations had the highest recorded mean root As concentrations in both locations (Table 3), yet in Renhua it had the lowest plaque concentration of As (Table 3). The root As concentrations for SY-122 grown in Lechang (52.0 mg·kg^−1^) and Renhua (48.6 mg·kg^−1^) were similar, which is in stark contrast to the 10-fold differences for the corresponding DCB-extracted As and soil As levels observed in this survey (Table 3). In this regard, although the As concentrations in soil and root surface for Lechang were significantly higher, there was not a significant difference for concentration of As inside the root, suggesting that As in rice does not directly depend on the total As concentration in the soil and root surface but may be due to other factors and uptake mechanisms.

Regardless of rice cultivars and locations, the As concentrations in soil and DCB extracts were much higher than As concentrations in rice plants, excepting rice root, whereas no obvious trend was observed between the As concentrations in soil, DCB extracts, and roots (Tables 1–3). Rice roots contained considerably higher concentrations of As compared to any other parts of the plant, regardless of soil As concentration and rice cultivars (Tables 1–3). The levels of root As were found to be on average 7.7 times higher than their corresponding straw, a trend which was maintained throughout several orders of magnitude in grain As. Previous researches [31], [41], [42] also observed that much more As accumulated in rice root than other parts. In the current study, the levels of As in straw were found to be, on average, 15.4 times higher than their corresponding grain samples.

Both straw As concentration and mean As straw/soil transfer factors (S^traw^/S_oil_ TFs) based on total As concentrations were highly variable between and/or within Renhua and Lechang locations (Table 2). Mean S^traw^/S_oil_ TFs for Renhua and Lechang were significantly different, with the values of 0.36 and 0.079, respectively (Table 2), and in both location, the highest and the lowest mean S^traw^/S_oil_ TFs were seen for SY-122 and SY-428, respectively (Table 3).

The range of grain/soil transfer factors (G^rain^/S_oil_ TFs) were 0.012–0.057 for Renhua, and 0.0020–0.0047 for Lechang. There was an over 7-fold difference in mean G^rain^/S_oil_ TFs between Renhua and Lechang respectively, probably related to the significant difference of soil As between the two locations (Table 1). Similar to S^traw^/S_oil_ TF, the G^rain^/S_oil_ TF for SY-122 was significantly higher compared to SY-428, regardless of location (Table 3). Mean As grain/straw transfer factors (G^rain^/S_traw_ TFs) ranged from 0.038 to 0.11, averaging 0.076, which was a little higher compared to the As G^rain^/S_traw_ TFs in rice surveyed in Guangdong [22]. Differences in As G^rain^/S_traw_ TFs were not apparent between locations (Table 1) and cultivars respectively (Tables 3).

### Factors affecting As transfer

**Figure 2:**
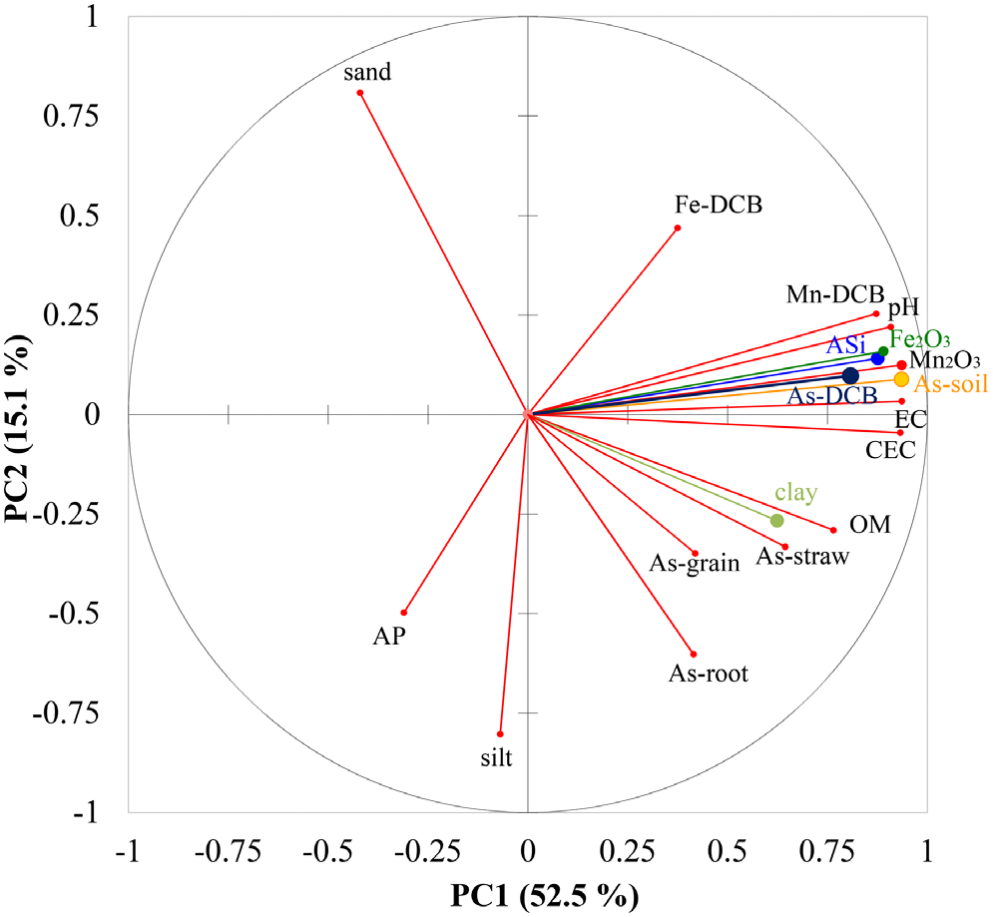
Plot of the first two principal components from Principal Component Analysis (PCA). The PCA was performed with As, Fe and Mn in DCB extracts, total As in soil, Fe_2_O_3_ and Mn_2_O_3_, available phosphorus (AP) and Si (ASi), selected soil properties, As in rice tissues.

As discussed above, As uptake by rice plants appears more affected by As availability than total As in the soil. The bioavailability of As to plants is governed by key edaphic physiochemical properties (e.g., pH, Eh, organic matter, texture, Fe/Mn-oxides/hydroxides, and phosphorus, silicon, and sulfur concentrations); environmental conditions and modification of the soil in the rhizosphere; these factors interact to influence As speciation in the soil [16], [41]. Rice is normally cultivated in flooded paddy soil, an environment that leads to a mobilization and, hence, a much enhanced bioavailability of As to rice plants. Rice is also a strong accumulator of the macro-nutrient silicon, an element that plays an important role in the defense against a range of biotic and abiotic stresses [10]. The principal component analysis (PCA) (Fig. 2) was performed with the concentrations of As, Fe and Mn in DCB extracts from root surfaces, the concentration of total As in soil, the concentrations of Fe and Mn oxides, AP and ASi, selected soil properties, and the concentrations of As in rice tissues in order to analyze the relationships among these indices and identify the factors affecting As transfer. The first 2 principal components accounted for 67.6% of the variability observed among all the cultivars and across all harvest sites. The results from PCA is in agreement with predictions that plant As is determined more strongly by external soil properties affecting As availability than by differences in internal plant processes.

The Fe and Mn oxide phases are common in various soils and are very efficient in sorbing As [15]. Manganese plaque and Fe and Mn oxides positively correlated with As in rice tissues respectively (Fig. 2), indicating Mn plaque and Fe and Mn oxides in soil may inhibit As transfer from soil to rice plant. There were 1.2 times, 12.2 times, and 27.4 times difference in iron plaque, manganese plaque, and Mn_2_O_3_ level respectively between Lechang and Renhua (Table 1), which might account for fact that the higher As in the Lechang soils did not result in significantly greater As in plant tissues compared with the rices grown in Renhua region.

The mechanism for arsenate uptake, the dominant inorganic As species under aerobic conditions, is through phosphate transporters, as arsenate is an analogue of phosphate [43]. Addition of phosphate to the soil may decrease arsenate uptake and consequently may reduce As toxicity, depending on soil conditions etc. [22]. In the current study, however, no remarkable relationships were observed between the AP in soil and rice As (Fig. 2), which is incompatible with the arsenate uptake mechanism discussed above, probably due to arsenite being the predominant form of As in flooded paddy soils [18], which doesn’t compete with phosphate for transport as arsenate. Arsenite shares the same transport system responsible for silicon uptake, both influx and efflux transporters mediate transport of arsenite [28], and arsenite associated with iron plaque may be much more easily desorbed than arsenate [15], [16]. Therefore, the application of silica fertilizer to soil can decrease the transfer of As from the soil and irrigation water to rice. The significantly higher available silicon in Lechang (p = 0.013), as shown in Table 1, may also be responsible for the fact that the higher soil As in Lechang did not result in significantly greater As in rice grain compared with the rices grown in Renhua region.

The PCA effects of root As, straw As, and grain As were close to each other, indicating the significant positive relationships between these rice tissues, while the As in rice tissues showed negative relationship with DCB-extracted As and soil As, respectively (Fig. 2). This further verifies that a rise in the soil As may not increase the accumulation of As in rice tissues.

Soil texture is another important factor affecting As bioavailability [15]. In general, soils with a clayey texture have less availability of As compared with sandy soils [17]. As observed in Table 1, the significant higher soil clay content in Lechang (p = 0.010) may decrease the availability of its soil As, so that even though these soils contained more As, they consequently inhibited its uptake by rice plants.

The solubility and bioavailability of As can be affected by soil pH because it controls the As speciation and leachability [15]. The soil pH differed significantly between Lechang and Rehua (p = 0.0096). The soils in Lechang ranged from neutral (pH 5.9) to slightly alkaline (pH 7.6) and in Renhua from strongly acidic (pH 4.4) to neutral (pH 6.0), respectively (Table 1). The soils collected from the Lechang mine region were expected to be more acidic and similar to those in Renhua. The industries in the northeast part of the paddy field (Fig. 1), including chemical plant, cement plant, textile mill, metal processing factory, plastic products factory, and bulb factory, might be affecting the soil pH. Arsenite solubility increases as the pH decreases within the range commonly found in soil (pH 3–9), while the pattern is reversed in the case of arsenate. Arsenite predominates in flooded paddy soils. In this regard, for the current study, a decline in soil pH can increase the mobilization of As in soils, which explain why we observed nearly equal concentrations of As in rice grain regardless of the soil As levels. The significant higher soil pH in Lechang may decrease the availability of its soil As.

By understanding the factors controlling bioavailability of As to rice plants and mechanisms of As uptake in plants, one could develop proper strategies for limiting As accumulation in rice grains. Possibilities include altering farm practices, e.g., growing rice aerobically in raised beds instead of in the traditional flooded paddy fields, which offers an opportunity to reduce the mobilization of soil arsenite and curtail As transfer from soil to grain. This approach would require, however, a fundamental change in farming practices in Asia [6], [14], and aerobically produced rice is generally lower yielding [16], [44] and more susceptible to rice blast disease and heat stress [45]. In addition, silica and phosphate fertilizations can be applied in soil to decrease As accumulation in rice, dependent on soil conditions [16]. Another tack would be the selection of rice cultivars with low accumulation of As in grains [40]. To be a success on the farm, any new cultivars will have to have decent yields. A hypothetical cancer risk pales in comparison with an empty stomach [6].

## Conclusions

The results indicated both environmental and genetic effects caused diversity for grain As concentration among different rice cultivars grown in two locations in China with mining-contaminated soils. All the grain samples in this study exceeded national food standards for grain As. The fact that grain As levels were not significantly different in the rices from the two areas differing significant for soil As levels, suggests that As uptake by rice is determined more by As availability rather than by total As in the soil. The As behaviour in the soil-rice system was found associated with various factors, i.e., iron and manganese plaque, iron and manganese oxides in the soil, soil available silicon and phosphorus, soil pH, soil organic matter, and soil texture. Understanding the mechanisms controlling As uptake would improve our understanding of how soil As sometimes but not always increases rice grain As, and to develop genetic and physico-chemical strategies for reducing As accumulation in rice grains.

## Supporting Information

Table S1 **The respective rice cultivar corresponding to the sampling sites.**
(DOCX)
