# Supplementary material for: Mechanisms Controlling Arsenic Uptake in Rice Grown in Mining Impacted Regions in South China
Source: PLoS One. 2014 Sep 24;9(9):e108300. doi: 10.1371/journal.pone.0108300 (PMC4177218; doi:10.1371/journal.pone.0108300)
Supplement: Table S1 — The respective rice cultivar corresponding to the sampling sites. (DOCX) [file pone.0108300.s001.docx]

**Table S1** The respective rice cultivar corresponding to the sampling sites

| Rice cultivar | Sampling sites in Renhua (n=20) | Sampling sites in Lechang (n=8) |
| --- | --- | --- |
| SY-428 | R1, R10, R16 | L1, L2, L5 |
| MBYZ | R5, R7 | L8 |
| MXZ | R6, R9, R11, R19 | - |
| TY-10 | R2, R4 | - |
| SY-162 | R3, R8, R18 | - |
| SY-86 | R15, R17 | - |
| SY-122 | R20 | L4 |
| DY-162 | R13 | - |
| JY-118 | R14 | - |
| SY-89 | R12 | - |
| SY-82 | - | L6, L7 |
| FY-998 | - | L3 |
